# Supplementary material for: Reconciling Mining with the Conservation of Cave Biodiversity: A Quantitative Baseline to Help Establish Conservation Priorities
Source: PLoS One. 2016 Dec 20;11(12):e0168348. doi: 10.1371/journal.pone.0168348 (PMC5173368; doi:10.1371/journal.pone.0168348)
Supplement: S1 Dataset — (ZIP) [file pone.0168348.s002.zip › Taxa/Serra Norte/SN_2013/N5S/Lista N5S-39.pdf]

CAVIDADE N5S-0039

Classe	Ordem	Fam/Outros	Gên/Outros	Espécie	S-U	U-S	Zon.
Arachnida	Acari	(Parasitengonina)		sp.2		X	E
Arachnida	Acari	Opilioacaridae		sp.1		X	E
Arachnida	Acari	Rhagidiidae		sp.3	X		E
Arachnida	Araneae	Ctenidae		jovem	1	7	E
Arachnida	Araneae	Drymusidae		jovem	X	X	E
Arachnida	Araneae	Filistatidae		jovem	X		E
Arachnida	Araneae	Ochyroceratidae		jovem		X	E
Arachnida	Araneae	Oonopidae	*Capitato*	sp.1		X	E
Arachnida	Araneae	Pholcidae	*Mesabolivar*	*aurantiacus*		X	E
Arachnida	Araneae	Pholcidae	*Mesabolivar*	sp.1		X	E
Arachnida	Araneae	Salticidae		jovem	X	X	E
Arachnida	Araneae	Scytodidae	*Scytodes*	jovem		1	E
Arachnida	Araneae	Theraphosidae		jovem		4	E
Arachnida	Araneae	Theridiidae	*Theridion*	sp.2	X	X	E
Arachnida	Araneae	Theridiosomatidae	*Plato*	sp.1		X	E
Arachnida	Opiliones	Cosmetidae	*Roquettea*	*singularis*	1		E
Arachnida	Pseudoscorpiones	Chernetidae	*Spelaeochernes*	sp.1	X	X	E
Arachnida	Pseudoscorpiones	Chthoniidae	*Pseudochthonius*	sp.1	X		E
Arachnida	Ricinulei	Ricinoididae	*Cryptocellus*	jovem		1	E
Chilopoda	Scutigeromorpha	Pselliodidae	*Sphendononema*	jovem		1	E
Diplopoda	Polydesmida	Chelodesmidae		sp.3		1	E
Entognatha	Collembola	Entomobryidae		sp.1	X	X	E
Entognatha	Collembola	Isotomidae		sp.1		X	E
Entognatha	Collembola	Paronellidae		sp.1	X	X	E
Entognatha	Diplura	Campodeidae		sp.1	X	X	E
Insecta	Blattodea	Blattidae		sp.4		5	E
Insecta	Blattodea	Blattidae		sp.5	1		E
Insecta	Coleoptera	Scydmaenidae		sp.2		X	E
Insecta	Coleoptera			jovem	X	X	E
Insecta	Diptera	Cecidomyiidae	Cecidomyiinae	sp.		X	E
Insecta	Diptera	Chloropidae		sp.		X	E
Insecta	Diptera	Phoridae	Metopininae	sp.		X	E
Insecta	Diptera	Psychodidae	Phlebotominae	sp.	X	X	E
Insecta	Heteroptera	Dipsocoroidea		jovem	X		E
Insecta	Heteroptera	Reduviidae	Emesinae	sp.2		1	E
Insecta	Heteroptera	Reduviidae	Reduviinae	sp.	1	4	E
Insecta	Homoptera	Cixiidae		jovem		X	E
Insecta	Hymenoptera	Formicidae	*Brachymyrmex*	sp.1	X		E
Insecta	Hymenoptera	Formicidae	*Brachymyrmex*	sp.2	X	X	E
Insecta	Hymenoptera	Formicidae	*Camponotus*	sp.2	X		E
Insecta	Hymenoptera	Formicidae	*Dolichoderus*	aff. *bispinosus*	X	X	E
Insecta	Hymenoptera	Formicidae	*Gnamptogenys*	sp.1		X	E
Insecta	Hymenoptera	Formicidae	*Hypoponera*	sp.1		X	E
Insecta	Hymenoptera	Formicidae	*Pachycondyla*	sp.1		X	E
Insecta	Hymenoptera	Formicidae	*Pachycondyla*	sp.2	X	X	E
Insecta	Hymenoptera	Formicidae	*Paratrechina*	sp.1	X	X	E
Insecta	Hymenoptera	Formicidae	*Pheidole*	sp.2		X	E
Insecta	Hymenoptera	Formicidae	*Pheidole*	sp.3		X	E
Insecta	Hymenoptera	Formicidae	*Pseudomyrmex*	aff. *opaciceps*		X	E

Insecta	Hymenoptera	Formicidae	*Solenopsis*	sp.1		X	E
Insecta	Hymenoptera	Formicidae	*Trachymyrmex*	sp.1	X		E
Insecta	Isoptera	Termitidae	*Embiratermes*	sp.		X	E
Insecta	Isoptera	Termitidae	*Nasutitermes*	sp.	X	X	E
Insecta	Lepidoptera	Gelechioidea		sp.		X	E
Insecta	Lepidoptera	Noctuoidea		sp.5		2	E
Insecta	Lepidoptera	Tineoidea		sp.1		X	E
Insecta	Lepidoptera	Tineoidea		sp.3		X	E
Insecta	Lepidoptera			jovem	X	X	E
Insecta	Neuroptera	Myrmeleontidae		jovem	X	X	E
Insecta	Orthoptera	Phalangopsidae	*Paraclodes*	sp.		3	E
Insecta	Orthoptera	Phalangopsidae	*Phalangopsis*	sp.		2	E
Insecta	Psocoptera			jovem	X	X	E
Malacostraca	Isopoda	Philosciidae		sp.1		X	E
Malacostraca	Isopoda	Philosciidae		sp.2		X	E
Amphibia	Anura	Strabomantidae	*Pristimantis*	cf. *fenestratus*	2		E
